# Rituximab induction therapy for de novo ANCA associated vasculitis in pregnancy: a case report

**DOI:** 10.1186/s12882-018-0949-7

**Published:** 2018-06-28

**Authors:** Claire Harris, Judith Marin, Monica C. Beaulieu

**Affiliations:** 1 Division of Nephrology, University of British Columbia, Vancouver, BC Canada; 2 St. Paul’s Hospital, 1081 Burrard St. Vancouver BC, Vancouver, Canada; 3 BC Provincial Renal Agency, Vancouver, BC Canada

**Keywords:** Antineutrophil cytoplasmic antibody (ANCA), Pregnancy, Case report, Rituximab, Myeloperoxidase (MPO), Vasculitis

## Abstract

**Background:**

The diagnosis of antineutrophil cytoplasmic antibody (ANCA) associated vasculitis (AAV) is rare in pregnancy but potentially life threatening. There are no randomized controlled trials to guide the management of AAV in pregnancy and fetal safety data remains limited. Rituximab administration, a treatment for AAV, has been reported in pregnant women with reassuring fetal outcomes in the oncology and rheumatology literature; however, no published reports describe its use in AAV.

**Case presentation:**

We present a case of de novo myeloperoxidase positive (MPO) AAV diagnosed at 22 weeks gestation. Clinical presentation included elevated serum creatinine at 177 μmol/L, hematuria and nephrotic range proteinuria along with high-titre MPO. Diagnosis was confirmed by renal biopsy. Patient was treated with methylprednisolone IV followed by oral prednisone 70 mg daily and Rituximab 650 mg IV weekly for four weeks followed by azathioprine maintenance therapy and prednisone taper. Delivery occurred at 29 weeks gestation via cesarean section for maternal neurologic symptoms concerning for preeclampsia. Maternal and fetal CD + 19 cells were depleted at time of delivery with associated fetal lymphopenia in the absence of infection or other complications related to Rituximab use. The patient experienced a reduction in proteinuria and inflammatory markers following Rituximab therapy; however, serum creatinine increased to 375 μmol/L by 11 weeks post-partum.

**Conclusion:**

We report the first use, to our knowledge, of Rituximab with corticosteroids for induction therapy of AAV in pregnancy.

## Background

The de novo diagnosis of antineutrophil cytoplasmic antibody (ANCA) associated vasculitis (AAV) is rare in pregnancy. Treatment of AAV in pregnancy poses a therapeutic dilemma as the risks of teratogenicity and adverse fetal outcomes are weighed against the potential fatal maternal outcomes of untreated severe disease. We report the first use, to our knowledge, of Rituximab with corticosteroids for induction therapy in MPO associated ANCA vasculitis with severe renal involvement diagnosed in the second trimester of pregnancy.

## Case presentation

A 20 year old gravida 1, para 0, female of Salvadoran descent was referred to nephrology for deteriorating kidney function and the nephrotic syndrome at 22 weeks gestational age (GA). Past medical history included a diagnosis of juvenile rheumatoid arthritis at age 12 treated with nonsteroidal anti-inflammatory drugs (NSAIDs) with resolution of symptoms into adulthood without therapy. The patient also endorsed pre-conception use of intranasal cocaine and cigarettes with no use (documented on urine drug screen) post conception. Medication use included prenatal vitamins, diclectin and acetaminophen. The patient reported a family history of rheumatoid arthritis diagnosed in her mother and maternal grandmother.

At presentation the patient’s serum creatinine was 177 μmol/L compared to a previous creatinine of 82 μmol/L three months earlier and a pre-pregnancy value of 56 μmol/L. She endorsed progressive lower limb edema beginning in early pregnancy. A history of tea coloured urine, epistaxis and sinus pressure was elicited. She did not have hemoptysis or rash but endorsed arthralgias in her shoulders, wrists, distal interphalangeal joints and ankles.

On examination her blood pressure was 116/60 mmHg. Relevant examination findings included pitting edema to the knees bilaterally. There were no active joints or rash.

24-h urine for protein yielded 9.81 g with > 100 RBC/hpf seen on microscopy. Urine albumin to creatinine ratio was 450 mg/mmol. Urine drug screen was negative for cocaine. Serum albumin was 20 g/L, antinuclear antibody 1:80, double stranded DNA negative, extractable nuclear antigen negative, C3, C4, normal and rheumatoid factor, cyclic citrullinated peptide negative, C-reactive protein 13 (normal < 10). HIV, Hepatitis B and C serology were negative. ANCA serology was positive with MPO antibody tire of 107.7 Antibody Index (AI) (normal < 20).

Renal ultrasound revealed structurally normal kidneys. Obstetrical ultrasound revealed mild intrauterine growth restriction (IUGR). Chest x-ray was unremarkable.

Renal biopsy was performed urgently with a diagnosis of pauci immune glomerulonephritis. Three of 14 glomeruli on light microscopy contained cellular crescents, 4 glomeruli fibrous crescents, 1 globally sclerosed. Up to six glomeruli contained segmental sclerosis with only five to 10 % interstitial fibrosis and tubular atrophy.

The patient was counselled regarding the maternal and fetal risks associated with her diagnosis and her therapeutic options including termination and elected to continue with the pregnancy. In particular, the risk of progression of renal impairment and potential requirement of dialysis during pregnancy were discussed, in addition to the high risk of preeclampsia and preterm labour if the pregnancy was continued. The patient was treated with methylprednisolone 1 g IV daily × 3 followed by oral prednisone 70 mg daily dosed by ideal weight and then Rituximab 650 mg IV weekly for four weeks. No infusion reactions to Rituximab were noted. The patient developed diffuse striae distensae which may have been a result of the high dose prednisone in the context of pregnancy and weight gain. Serum creatinine continued to rise throughout pregnancy to 250 μmol/L, despite a reduction in proteinuria to a urine albumin creatinine ratio of 125 mg/mmol and improvement in symptoms. At 27 weeks GA, the patient became hypertensive necessitating use of labetalol therapy. She was initiated on erythropoietin for anemia. The patient developed thrombocytopenia which was felt to be immune mediated in nature with a nadir of 64. Repeat obstetrical ultrasounds revealed normalization of fetal growth.

Delivery occurred at 29 weeks GA via caesarean section for worsening maternal headache and visual disturbance concerning for preeclampsia and worsening renal function with serum creatinine of 275 μmol/L. Maternal CD19+ cells were depleted at time of delivery. Neonatal birth weight was 1010 g and the neonate spent 10 weeks in the neonatal intensive care unit before discharge in healthy condition. Neonatal CD19+ cell levels were depleted at birth with associated transient lymphopenia without infectious complications. After delivery, the patient’s creatinine rose to a value of 375 μmol/L (eGFR 14 ml/min) at 11 weeks post partum. Prednisone was tapered slowly to 10 mg daily and the patient was initiated on azathioprine maintenance therapy four months after Rituximab induction and titrated up to a dose of 2 mg/kg/day. At the time of maintenance therapy initiation, the MPO titer was 5.8 Units (normal < 1).

## Discussion

Without specific treatment ANCA associated vasculitis is associated with high mortality and poor renal outcomes. The most recent KDIGO guidelines suggest the use of cyclophosphamide and corticosteroids as induction treatment with grade 1A evidence [1]. Rituximab and corticosteroids may be used as an alternative initial treatment in patients without severe disease or in whom cyclophosphamide is contraindicated (1B) [1]. The evidence for treatment of ANCA associated vasculitis in pregnancy is limited to case report and case series data.

Renal dysfunction is a well-established risk factor for pregnancy complications including preeclampsia, preterm labour and pregnancy loss [2]. It is generally accepted that active autoimmune disease such as vasculitis in pregnancy further increase these risks but the literature is limited to case reports in this area and thus attributable risk is unknown [3].

The literature describing ANCA associated vasculitis in pregnancy is heterogeneous and is dominated by descriptions of patients with known ANCA vasculitides in remission prior to conception. In one case study, Presta et al. reported the pre-conception use of plasma exchange, cyclophosphamide and prednisone for MPO-ANCA vasculitis 1 month prior to unplanned conception and there was no fetal complications [4].

De novo ANCA associated vasculitis in pregnancy is rare. A case series of 12 patients with de novo ANCA associated vasculitis in pregnancy was reported by Alfhaily et al. in 2009 [5]. Treatment regimens included combinations of prednisone plus cyclophosphamide, Intravenous immunoglobulin (IVIG) or azathioprine. One fetal death was reported, 2 pregnancies were terminated for medical indications with a mean live birth gestational age of 34 weeks and good fetal outcomes. One patient developed pre-eclampsia. There was one maternal death in this series with the majority of women achieving clinical remission.

Rituximab is listed as a Food and Drug Administration (FDA) category C drug per the previous FDA drug classification system [6]. As an IgG isotype antibody, it crosses the placenta beginning at 16 weeks gestation [7]. Pregnancy outcomes after maternal exposure to rituximab have been described in primarily in the oncology literature with some reports of use in autoimmune diseases including rheumatoid arthritis and systemic lupus erythematosus. A review of Rituximab exposure during 9 pregnancies for oncology (6 lymphoma) and autoimmune (1 idiopathic thrombocytopenic purpura, 1 thrombotic thrombocytopenic purpura, 1 autoimmune hemolytic anemia) indications reported no serious neonatal outcomes [6]. Although B cell depletion occurred in 4/5 neonates tested during the first week of life, recovery of B cell populations occurred by 6 months and no serious infections were reported even in the 2 neonates with low white blood cell counts at birth. A possible explanation for the absence of infections reported thus far in the literature is that neonates depend on maternal IgG for immunity during the first few months of life while B-cell function still developing during this time frame even in unexposed normal neonates [8].

A case series by Chakravarty reviewed 153 pregnancies with maternal exposure to rituximab and 90 live births including 21 pregnancies with antenatal exposure [9]. The rate of preterm labor was 19%, compared to 10–12% in the general population however this rate is comparable to women with chronic medical diseases. The rate of congenital malformations was the same as the general population. The rate of miscarriage was higher possibly due to frequent testing detecting early pregnancies or again due to maternal comorbidities. Four neonatal infections occurred but no pattern was identified regarding timing of rituximab exposure and no infections occurred in the 7 neonates who developed cytopenias.

With respect to the use of rituximab for AAV prior to conception, Pendergraft et al. reported maternal and fetal outcomes in patients who received rituximab pre conception [10]. This retrospective analysis reported 8 pregnancies in 6 women with vasculitis who received rituximab prior to conception. Mean rituximab exposure was 5.2 months prior to estimated date of confinement (EDC), with 4 of 8 pregnancies conceived within one month of most recent rituximab infusion. Rituximab was discontinued at time of pregnancy diagnosis and patients were maintained on prednisone and/or azathioprine. There was one fetal demise due to a congenital anomaly unrelated to rituximab and maternal disease activity was quiescent in all but one pregnancy where prednisone was up titrated due to respiratory symptoms. At delivery 6 of 8 women had undetectable CD20+ B lymphocytes however 3 of 3 of fetal samples had present CD20+ B lymphocytes. Mean delivery occurred at 38 wks GA for the 7 live births.

Review of the literature reveals one prior case report of the use of rituximab for induction therapy in a case of de novo pauci immune glomerulonephritis which was ANCA negative [11]. The pregnancy was ended with termination and patient was switched to cyclophosphamide after receiving two doses of rituximab in addition to corticosteroids and plasmapheresis, therefore no neonatal outcomes are reported.

As a result of this case, we report the first known use of rituximab for induction therapy of ANCA associated vasculitis during an established pregnancy with continuation of the pregnancy to delivery. Although Rituximab exposure in the second and third trimester appears to cause neonatal B cell depletion this does not appear to be associated with adverse events in the neonate. Rituximab exposure does not appear to increase congenital malformations even when exposure occurs in the first trimester or just prior to conception. Further investigation is still required to determine the adequacy of the immune response to vaccination in the offspring and it is recommended that live vaccines should be avoided during the first 6 months of life if exposure occurred after the first trimester [12].

## Conclusion

Although more evidence is required, Rituximab can be considered for the treatment of ANCA vasculitis during pregnancy. The decision to use any immunosuppressive medication in pregnancy requires careful counselling of the patient regarding the limitations of our knowledge in this area and the possible expected fetal outcomes. However, the importance of adequate treatment of a potentially life threatening maternal condition must be highlighted. Our patient developed preeclampsia and premature delivery even with improvement in markers of her vasculitis with rituximab therapy. This outcome emphasizes the high risk nature of pregnancies in patients with severe renal impairment and systemic inflammatory conditions and the need for specialized obstetrical care from a highly trained interprofessional team.

## Abbreviations

AAV: ANCA associated vasculitis
AI: Antibody Index
ANCA: Antineutrophil cytoplasmic antibody
EDC: Estimated date of confinement
FDA: Food and Drug Administration
GA: Gestational Age
IUGRs: Mild intrauterine growth restriction
IVIG: Intravenous immunoglobulin
MPO: Myeloperoxidase
NSAIDs: Nonsteroidal anti-inflammatory drugs
